# Supplementary material for: Tunable and high-purity room temperature single-photon emission from atomic defects in hexagonal boron nitride
Source: Nat Commun. 2017 Sep 26;8:705. doi: 10.1038/s41467-017-00810-2 (PMC5615041; doi:10.1038/s41467-017-00810-2)
Supplement: Supplementary file 1 — Supplementary Information [file 41467_2017_810_MOESM1_ESM.pdf]

SUPPLEMENTARY INFORMATION

Supplementary Note 1: Effects of the He^+ irradiation on exfoliated hBN flakes

Different ion doses have been investigated on hBN flakes with diverse thickness. Supplementary Fig.1a shows the optical characterization of a flake before the treatment (I), after the irradiation (II) and after the annealing (III). Similarly to what observed in TMDs semiconductors, pristine hBN shows PL emission mainly from the flake edges and cracks. The same flake is irradiated with three different doses. Region A with $8{\times}10^{13}$ ions cm^{-2}; Region B with $5{\times}10^{15}$ ions cm^{-2}; Region C with $1{\times}10^{17}$ ions cm^{-2}. All regions show high photoluminescence intensity before annealing due to ionization of individual atoms, lattice displacements, creation of vacancies and interstitials. Defects of the crystal structure induced by ballistic collisions with He ions cause the breakup of covalent bonds that can show fluorescence activities. Luminescence is observed only if the electrons free from covalent bonds have orbitals separated by an energy gap smaller than the exciting laser energy at 532 nm. Interestingly, the region with lower dose (A) shows the strongest fluorescence intensity. After annealing, the fluorescence from the irradiated areas is suppressed due to recrystallization and the effects of the treatment are visible only for the area irradiated with dose higher than 10^{15} ions cm^{-2}. Supplementary Fig.1b shows a magnified cutout of these regions. Several defects are visible inside region B and most of them are experimentally attributed to single photon emitters. Supplementary Fig.1c is an optical microscope image of the hBN flake after the full treatment. With a magnification of 40X the effects of FIB are already visible for the region B. The FIB area presents slightly darker color, probably due to the increase of the flake thickness. Supplementary Fig.1d shows the dependence on the ion dose of the intensity ratio between the areas outside and inside the irradiated regions. The values of the PL intensity are extracted from the PL maps with integration over an area of approximately 25 μm^2. This plot indicates an improvement in the intensity contrast between treated and untreated regions as the ion dose is increased.

Ion irradiation with a dose of $5{\times}10^{15}$ ions cm^{-2} has been tested on flakes with different thickness yielding similar results to the one shown in Fig.2 in the main text. Supplementary Fig.2 and Supplementary Fig.3 exemplify the effects on flakes with thickness of 70 nm and 30 nm, respectively. Similar swelling is

observed in almost all the investigated flakes and it seems to be independent from the hBN thickness as shown in Supplementary Fig.2a-c and Supplementary Fig.3a-c. Optical experiments confirms the reduction of the broadband emission inside the irradiated areas and the isolation of several point defects. Single photon emission from these defects is demonstrated with autocorrelation measurements performed with CW excitation. Examples of antibunching for two emitters (indicated with dotted yellow circles in the PL maps of Supplementary Fig.2b and Supplementary Fig.3b) are shown in Supplementary Fig.2d and Supplementary Fig.3d.

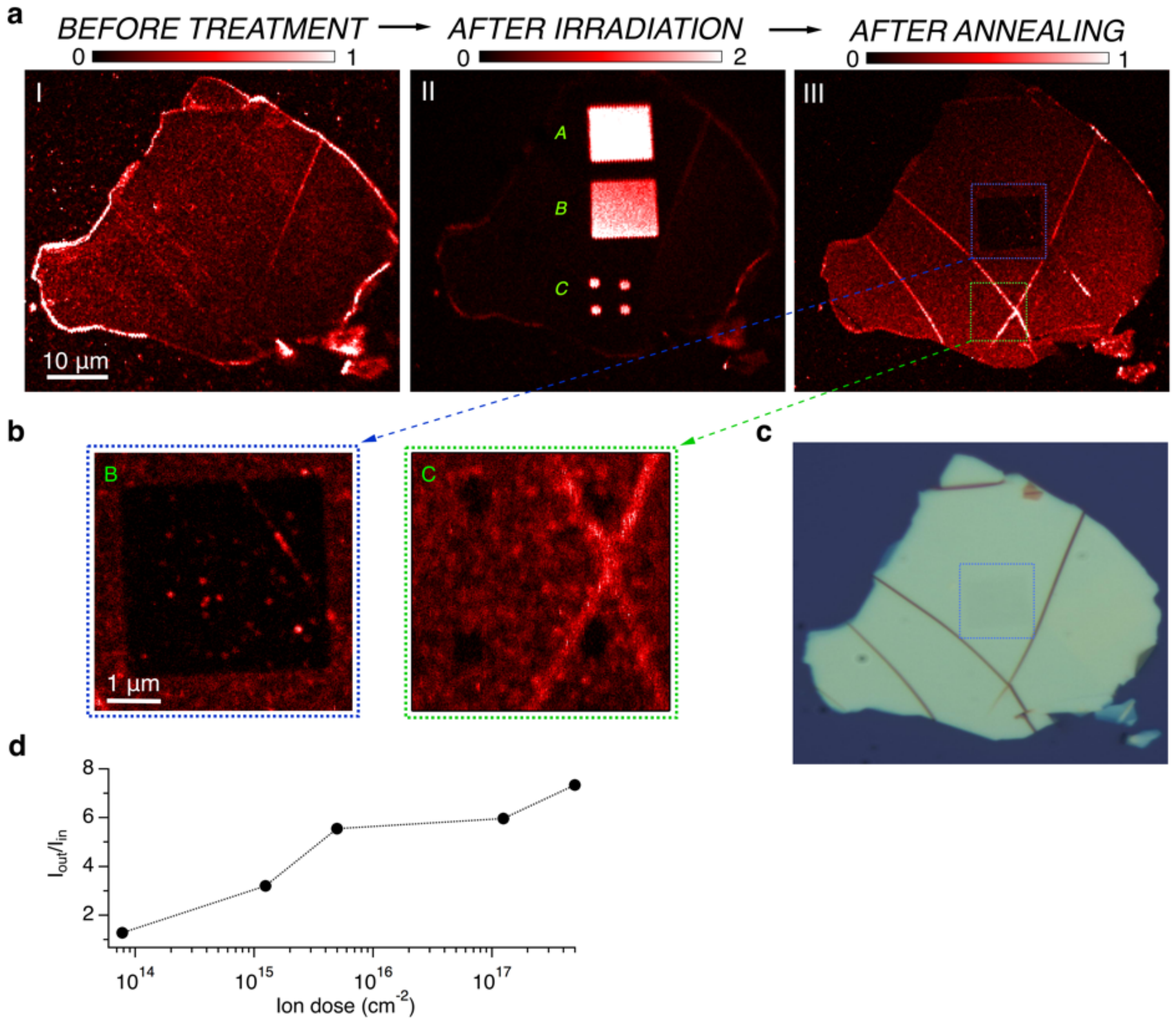

Supplementary Figure 1: **Effects of irradiation with different ion dose:** ***a*** - Images show the PL intensity map of a hBN flake before the treatment (I), after the He ion irradiation (II) and after the annealing (III). Three areas are irradiated with different dose: Region A with 8×10^{13} ions cm^{-2}; Region B with 5×10^{15} ions cm^{-2}; Region C with 10^{17} ions cm^{-2}. The maximum of the color scale is increased in panel II to highlight the different intensity between region A and B. ***b*** - Zoom of region B and C. Region B shows several SPEs within the irradiated area. ***c*** - Microscope image of the same flake taken with 40X magnification after the complete treatment. The effect of the irradiation with dose 5×10^{15} ions cm^{-2} is visible due to darkening of the flake color. ***d*** - Ratio of the PL intensity outside and inside the irradiated areas as a function of the ion dose. Intensity counts are integrated over areas of ~25 μm^2.

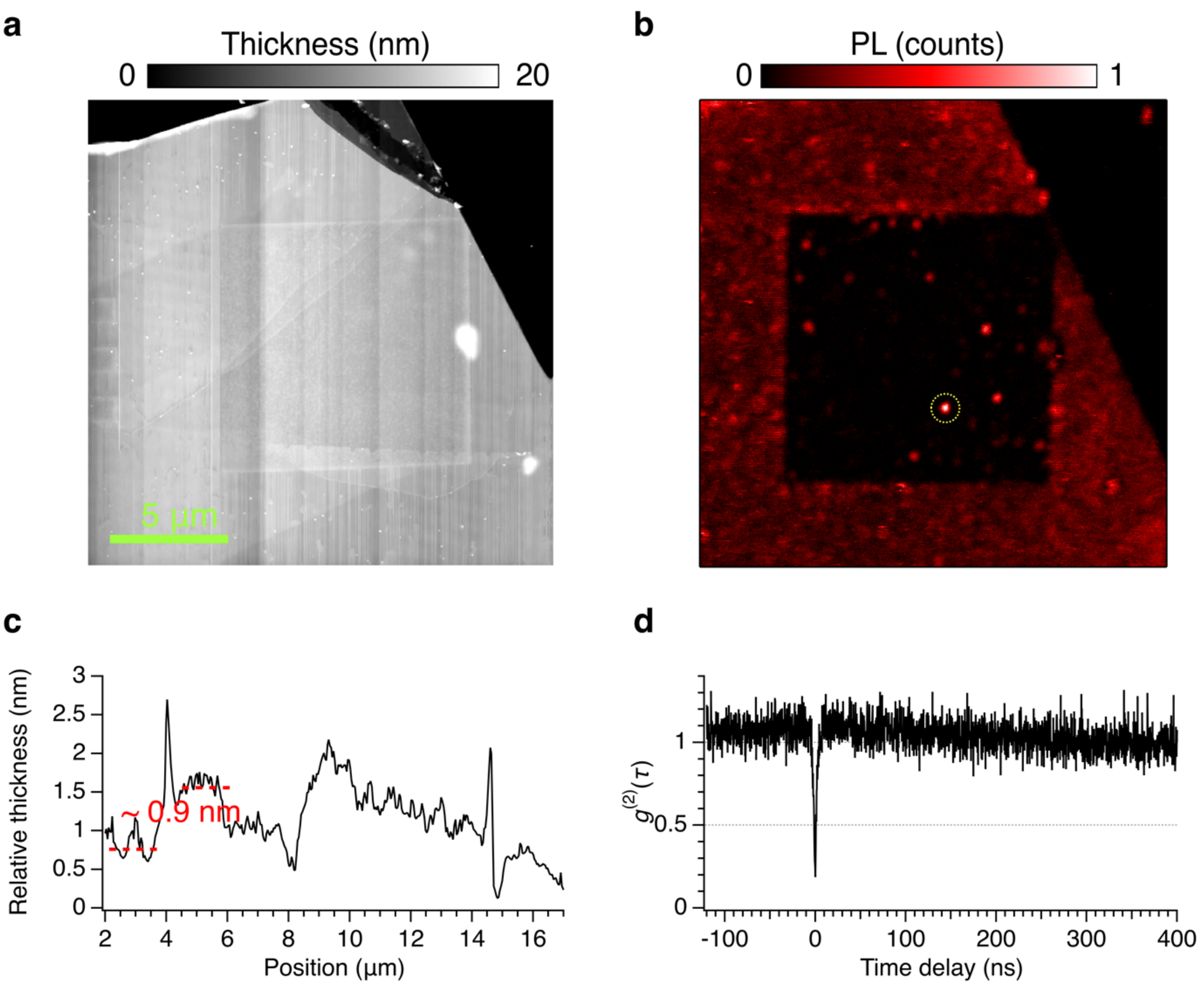

Supplementary Figure 2: **Effects of ion irradiation on a 70nm-thick hBN flake:** ***a*** - Topographic map of the implanted region measured with AFM. ***b*** - PL intensity map of the implanted region showing the reduction of the background intensity and several SPEs. ***c*** - Relative thickness calculated by integrating a vertical area of 5 μm width of the topographic map in *a*. ***d*** - Example of second-order correlation function ($g^{(2)}(\tau)$) with CW excitation of the emitter inside the irradiated area highlighted with a yellow circle.

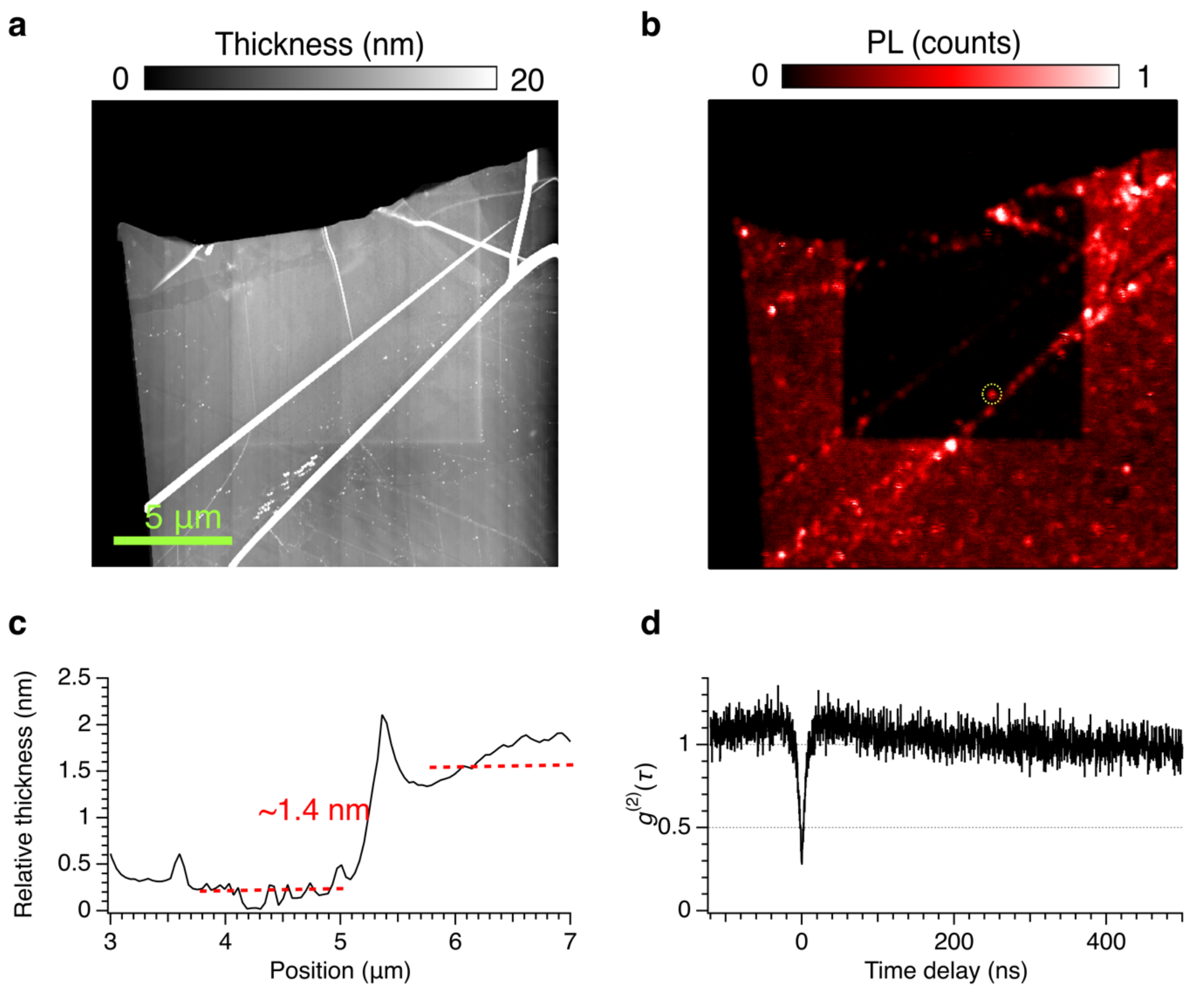

Supplementary Figure 3: **Effects of ion irradiation on a 20nm-thick hBN flake: *a*** - Topographic map of the implanted region measured with AFM. ***b*** - PL intensity map of the implanted region showing the reduction of the background intensity and several SPEs. ***c*** - Relative thickness calculated by integrating a horizontal area of 5 µm width of image *a*. ***d*** - Example of second-order correlation function ($g^{(2)}(\tau)$) with CW excitation of the emitter inside the irradiated area highlighted with a yellow circle.

Supplementary Note 2: Spectroscopy on single photon emitters

Supplementary Fig.4 shows the experiment setup for confocal photoluminescence measurements. Continuous wave or pulsed laser beam passes through the image relay system with scanning mirrors to be focused on the sample surface. In the 4f configuration, the galvanometer mirror scanner is placed at the focal plane of the first lens such that the beam angle can be precisely controlled. An angle variation at optical plane of the galvanometer mirrors corresponds to a different spatial position on the sample, allowing a precise control on the spatial position of the excitation and detection of individual defects.

In the detection path, the fluorescence photons from the emitters can be selectively routed towards avalanche photodiodes (APDs) or to a spectrometer by simply rotating a half wave plate (HWP) in front of polarized beam splitter (PBS). Second-order autocorrelation functions ($g^{(2)}(\tau)$) is measured by a Hanbury Brown and Twiss (HBT) interferometry setup using two APDs.

Supplementary Fig.5 shows a comparison between a single photon emitter inside and outside the irradiated region, similarly to the results already shown in Fig.3 in the main text. Supplementary Fig.5 is the PL intensity map of the region of interest. Several defects are visible inside the irradiated area, mainly at the cracks of the flakes. Two quantum emitters are analyzed and are highlighted by green (inside FIB area) and blue (outside FIB area) arrows. Supplementary Fig.5b is the autocorrelation measurement of the two emitters with CW excitation. Clearly the single photon purity ($g^{(2)}(0)$) is enhanced for the emitter inside the irradiated area. Solid lines in Supplementary Fig.5c, d show the spectra of these two emitters. Dashed lines indicate the background emission measured just next to the emitters. The spectral weight of the background for the not irradiated region is sensibly greater than for the irradiated region. The emitter inside the FIB area (Supplementary Fig.5c) provides a different example of spectral shape for the single photon emitters in layered hBN.

Background reduction due to He ion irradiation is observable also with Raman spectroscopy. This has been performed with low resolution grating, 100 lines mm^{-1}. Supplementary Fig.6 shows the spectra for the pristine (black line) and irradiated (blue line) hBN. Both spectra show a peak around 1000 cm^{-1}

attributed to the SiO_2 substrate. Overall, the not irradiated regions have stronger broad band emission than the irradiated regions.

Spectroscopy on single photon emitters has been performed also at cryogenic temperature. A comparison of the spectra of the same emitter for T = 300 K and T = 10 K is shown in Supplementary Fig.7 with black and red colors, respectively. The minimum linewidth of the ZPL emission measured at cryogenic temperature is ~0.7 nm.

Second-order correlation function is measured for different pump powers yielding good antibunching with $g^{(2)}(0) < 0.5$ up to $6{\times}10^6$ counts s^{-1}. Supplementary Fig.8 shows the autocorrelation functions for CW excitations at different pump powers. The curves have an increasing y-axis offset with steps of 0.5. Curves are fitted with the function $g^{(2)}(\tau) = 1 - (1 + a)\mathrm{e}^{-\frac{|\tau|}{\tau_1}} + a\mathrm{e}^{-\frac{|\tau|}{\tau_2}}$, where parameters τ_1 and τ_2 are the lifetimes of the excited and metastable states, respectively, while a is the fitting parameter.

The values of the autocorrelation functions at zero time delay are reported in Supplementary Fig.3c. Measurements of autocorrelation function with CW excitation yield slightly higher values of $g^{(2)}(0)$ than pulsed excitation. This mismatch is caused by the APD jitter that becomes negligible with short pulsed excitation.

Supplementary Fig.9 shows the saturation curve of the emitter reported in Fig.3 compared with the background emission measured inside the irradiated area. Background photon emission shows a linear behavior, as expected, and the intensity at the maximum excitation power ($P = 500\ \mu\mathrm{W}$) is 0.3 Mcps. For comparison, the emission rate of the emitter at the same power is 8.8 Mcps. The results of the fitting for the emitter and background emission intensity are reported in the legend.

Emitter lifetime is measured with a pulsed laser of 300 ps width. The result is shown in Supplementary Fig.10 and a single exponential fitting yields a lifetime of 2.4 ns.

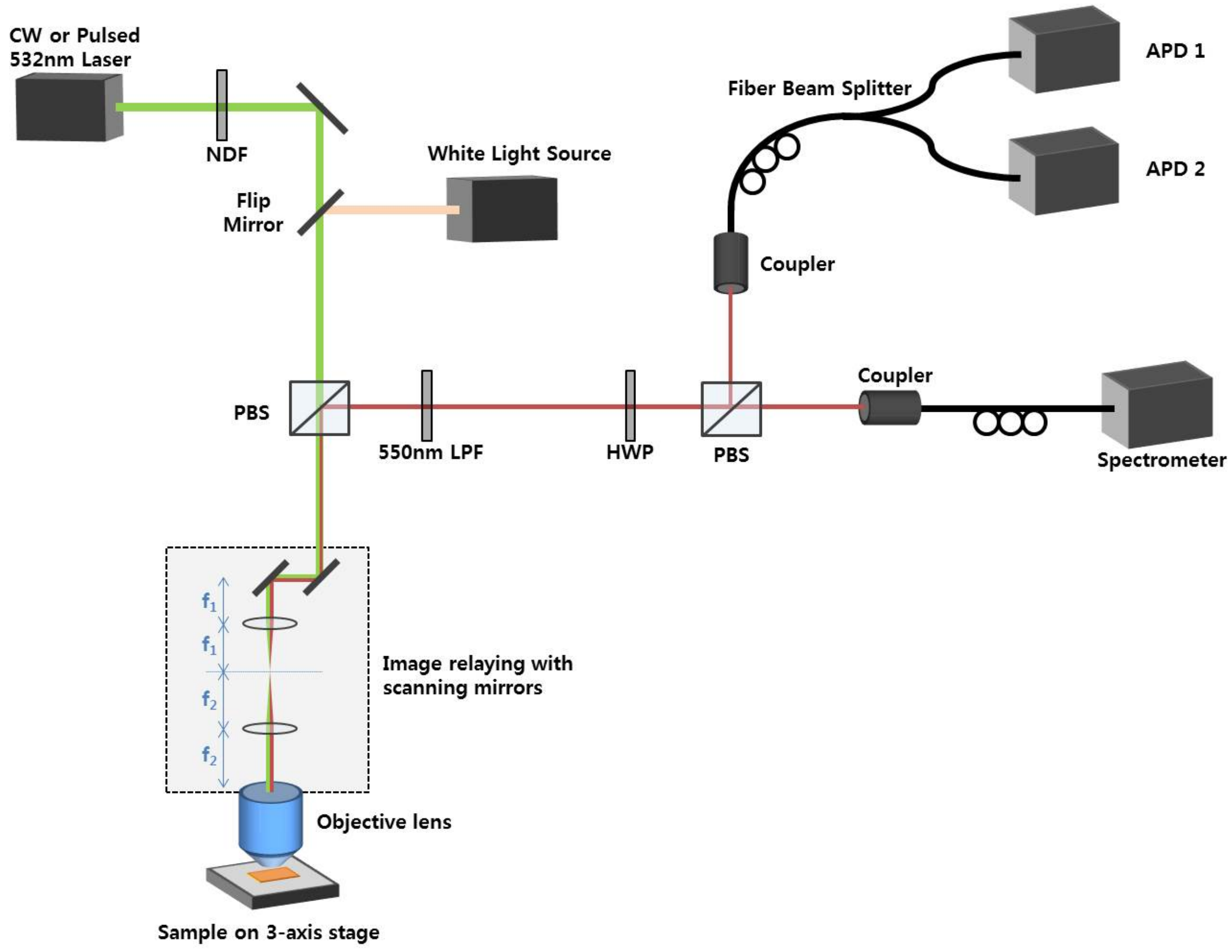

Supplementary Figure 4: **Confocal photoluminescence setup.** In the excitation path, continuous wave (CW) or pulsed laser is focused on the sample after passing through the image relay system. A galvanometer mirror system is placed at the focal plane of the first lens in a 4f configuration. Different angles of the beam after the galvanometer mirrors result in different spatial position on the sample surface. In the detection path the fluorescence photons are routed through a polarizer beam splitter (PBS) towards the APDs or the spectrometer. Second-order autocorrelation function ($g^{(2)}(\tau)$) is measured by a Hanbury Brown and Twiss (HBT) interferometry setup when the fluorescence goes into two separated APDs. White light is used to image the sample and locate the hBN flake on the SiO_2 surface. Legend: CW: Continuous wave, NDF: Neutral density filter, PBS: Polarization beam splitter, LPF: Long pass filter, HWP: Half wave plate.

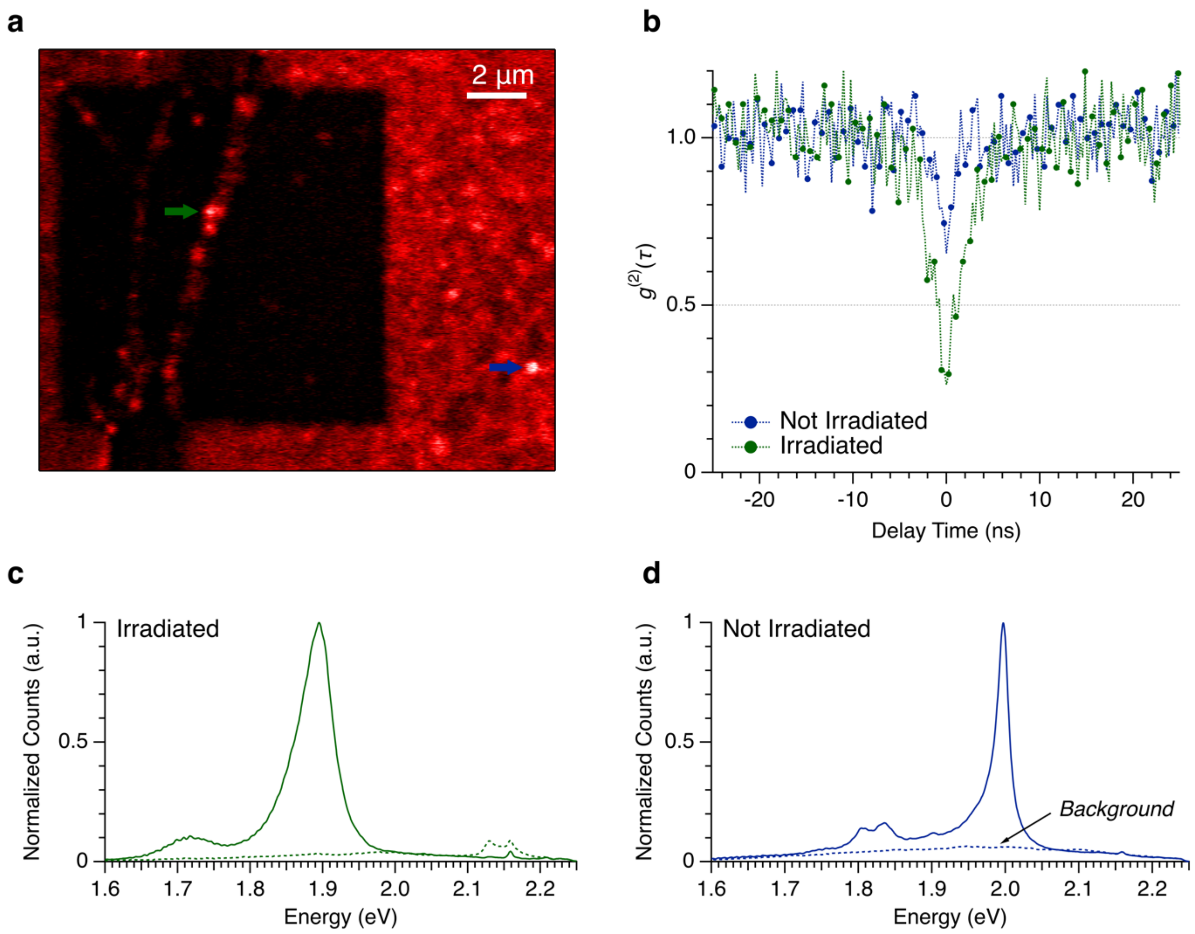

Supplementary Figure 5: **Increased purity of single photon emission.** ***a*** - The image shows the PL intensity map of a hBN flakes irradiated He ions with a dose of 10^{15} ions cm^{-2} in an area of 10 by 10 μm². Several point defects are visible inside this area, mainly in coincidence with cracks of the flake. Two quantum emitters are considered and are highlighted with a green (inside FIB area) and a blue (outside FIB area) arrow. ***b*** - Autocorrelation histogram recorded with CW excitation for the two emitters pointed in *a*. Dots show the data points and dashed lines serve to guide the eye. ***c, d*** - Emission spectra of the emitter inside (*c*) and outside (*d*) the irradiated area. The background spectrum is measured just next to the emitter and is shown with dashed lines.

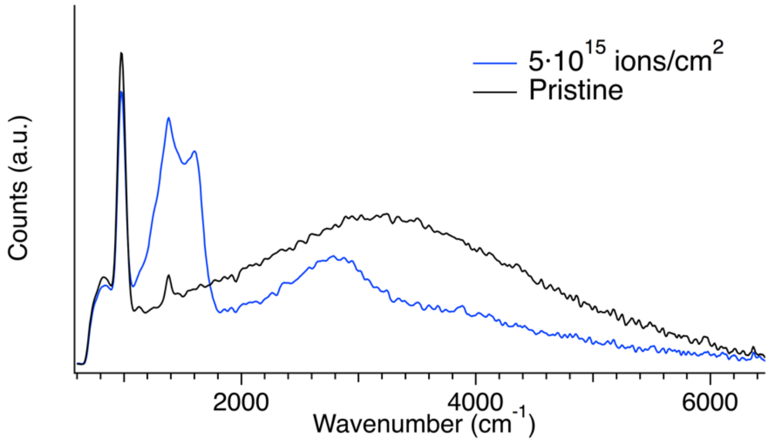

Supplementary Figure 6: **Low resolution Raman spectrum.** The plot shows the Raman spectra with low resolution grating (100 lines mm^{-1}) for pristine (black line) and irradiated (blue line) hBN. A Raman peak around 1000 cm^{-1} is observed in both cases and is associated to the SiO_2 substrate.

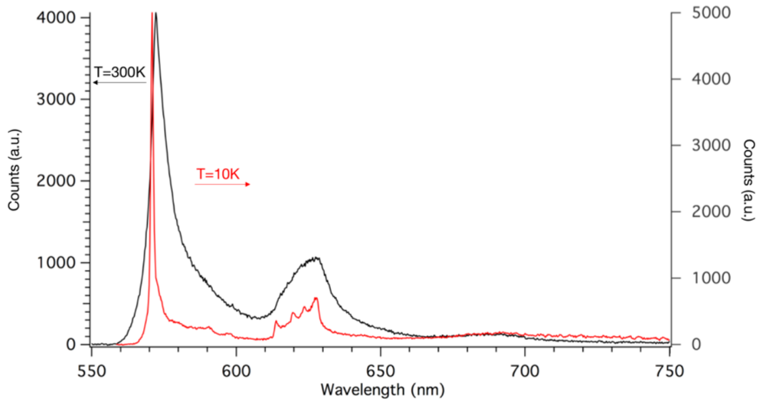

Supplementary Figure 7: **Temperature comparison.** The plot shows the spectra of a single photon emitter at room temperature (black line and left axis) and at T = 10 K (red line and right axis).

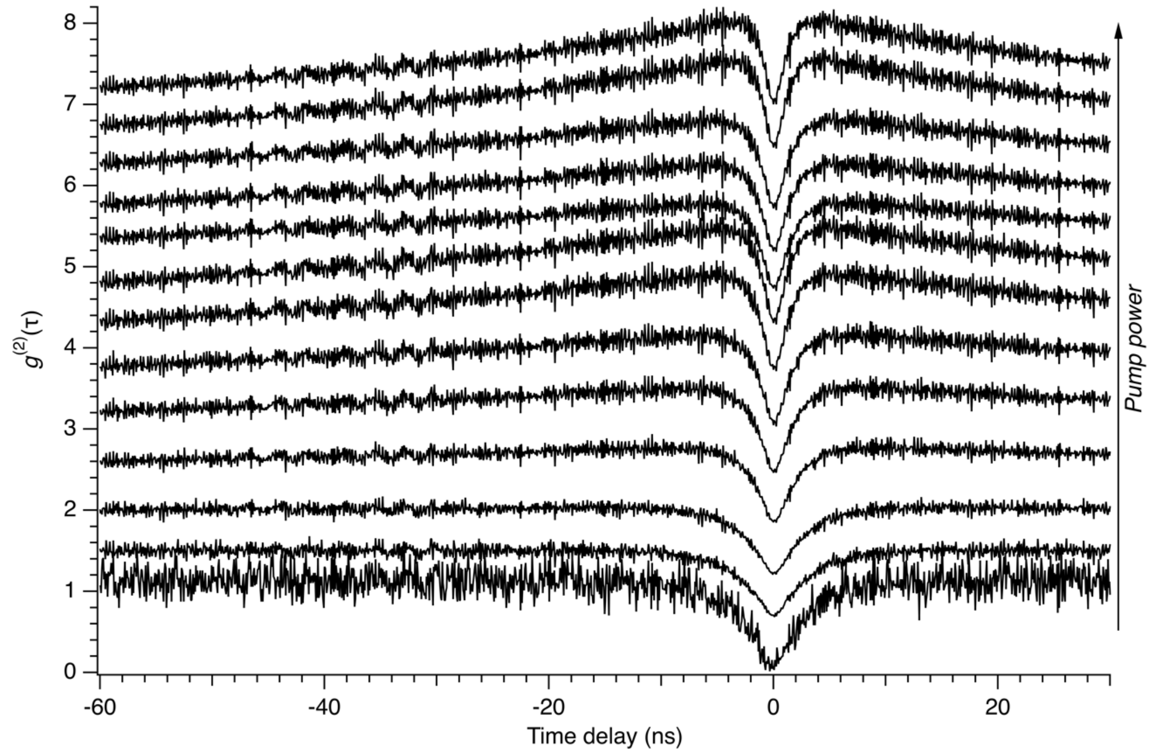

Supplementary Figure 8: **Second-order correlation functions as a function of pump power.** The plot shows the autocorrelation functions measured with CW excitation from which the values of $g^{(2)}(0)$ of Fig.3 are extracted. The pump power goes from 1 μW to 600 μW. For the sake of visibility, the graphs are shown with an increasing offset of the y axis of 0.5. At higher pump powers the autocorrelation functions exhibit the typical shape of a three-level single photon emitter due to the presence of a long living metastable state.

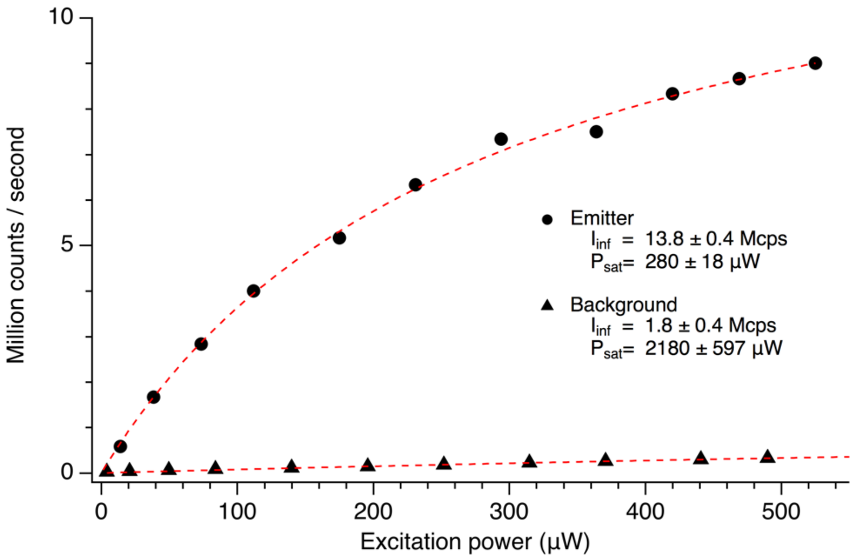

Supplementary Figure 9: **Emission rate as function of pump power.** The plot shows the same saturation curve of Fig.3. The emission from the background, shown in black triangles, exhibits a linear increase as a function of the excitation power. The legend reports the results of the fitting curves shown with red dashed lines.

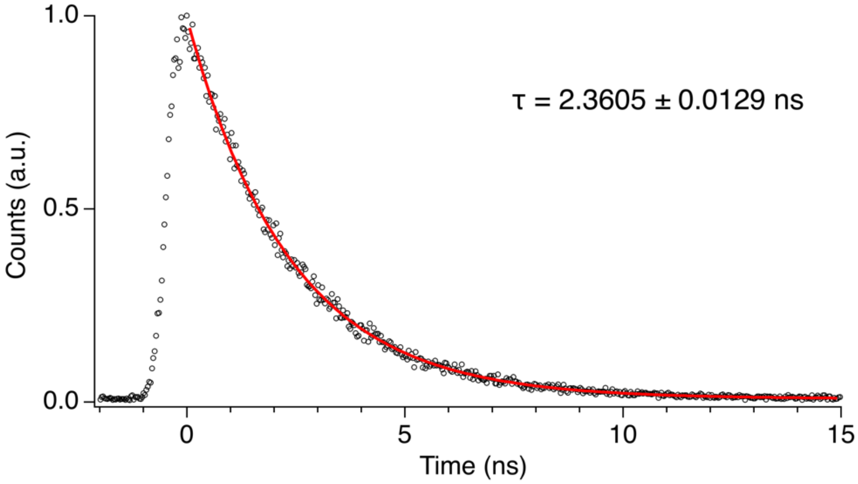

Supplementary Figure 10: **Lifetime measurement.** Normalized single photon emission as a function of time. A single exponential fitting yields a lifetime of ~2.4 ns.

Supplementary Note 3: Effect of sample transfer and strain on the spectral emission

Supplementary Fig.11 compares microscope image, PL intensity map, and spectrum before (left side: *a, c, e*) and after (right side: *b, d, f*) the sample transfer process. The transfer technique is described in detail in the Methods section. Microscope images show that most of hBN flakes are successfully transferred onto PC substrate. Supplementary Fig.11c, d are the photoluminescence measurements of He ion irradiated area of the flakes indicating that most of the point defects (bright spots) remain after the transfer. Dotted circles highlight a single photon emitter whose spectrum is shown in Supplementary Fig.11e, f. Background is slightly increased after the transfer due to the fluorescence of the PMMA film and the PC substrate, but it is still negligible when compared to the peak intensity of the emitter.

After the transfer, the PC substrate is mounted in the experiment setup as shown in Supplementary Fig.12. One side is clamped tightly and the other side is pushed downwards or pulled upwards to induce tensile or compressive strain, respectively. The amount of strain on the flake is calculated by the Euler-Bernoulli beam equation, which relates the beam deflection *z(x)* at position *x* to the applied load *q(x)*:

$$q(x) = \frac{d^2}{dx^2}\left(EI\,\frac{d^2 z(x)}{dx^2}\right) \qquad (1)$$

where *E* is Young's modulus and *I* is the second moment of area. In our rectangle-shaped substrate, $I = wh^3/12$, where *w* is the width and *h* is the height of the substrate. By assuming a point load $q = -P \times \delta(L)$ at position *x*=*L*, and with proper boundary conditions for fixed and free ends, we obtain $z(x) = -\frac{Px^2}{6EI}(3L - x)$. In a low bending regime, curvature reads $\varkappa = \frac{\mathrm{d}^2 z}{\mathrm{d}x^2}$ and strain $\varepsilon = -\varkappa h/2$. With this definition of deflection $\delta = z(L) = -\frac{PL^3}{3EI}$, the strain any arbitrary condition is given by $\varepsilon(x) = \frac{3h}{2L^3}\delta(L - d)$.

Supplementary Fig.13 shows another example of the effect of strain on the spectral emission. This emitter has a ZPL around 2.1 eV and blueshifts as the strain intensity is increased. This emitter has a tunability of +3.3 meV/%.

Simulations with various conditions of strain are shown in Supplementary Fig.13. The energy shift of the ZPL for different strain directions produce distinct behaviors, both in direction and magnitude of the energy shift. In particular, strain along ZZ1 and AC1 results a quadratic shift. Therefore, this simulation confirms our assumption about the non-monotonic behavior of the ZPL energy under the effect of strain. On the other hand, strain along ZZ2 and AC2 produces an almost linear shift. Biaxial strain is also investigated with homogeneous strain applied along the orthogonal directions AC2 and ZZ1. Biaxial strain also produces a quadratic energy shift. The effect of the high Poisson's ratio of the bendable substrate used for the experiments is taken into account and the simulation results in the curves labeled with ZZ1-P and

AC2-P in Supplementary Fig.14. ZZ1-P is the energy shift simulated when tensile strain is applied along the ZZ1 direction and an inhomogeneous strain of $-0.37\times\varepsilon_{\mathrm{ZZ1}}$ occurs along AC2. AC2-P indicates the case in which tensile strain is applied along AC2 and an inhomogeneous strain of $-0.37\times\varepsilon_{\mathrm{AC2}}$ occurs because of the Poisson's ratio.

Interestingly, the overall energy shift of the ZPL along all the directions covers an energy band of approximately 600 meV that justifies the spectral distribution of 300 meV observed experimentally.

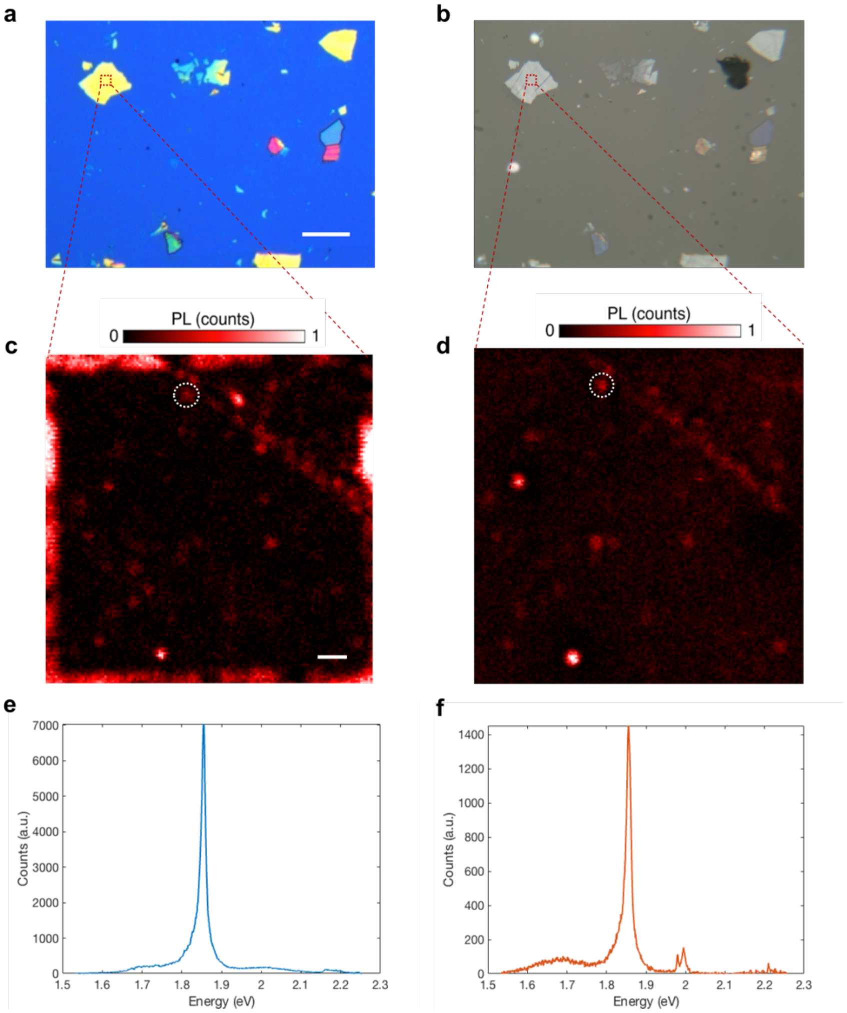

Supplementary Figure 11: **SPEs before and after the transfer process:** ***a, b*** - Microscope images of the hBN flakes before and after the transfer process onto the PC substrate. Almost all flakes are successfully transferred. Scale bar is 50 μm. ***c, d*** - PL maps of the sample before and after the transfer process. Scale bar is 1 μm. Almost all the point defects remain after the transfer. ***e, f*** - Example spectrum of the SPE highlighted with the dotted circle in *c* and *d,* before and after the transfer process. The small peaks around 2 eV in *f* are originated from Raman vibrations of the PMMA film covering the hBN sample.

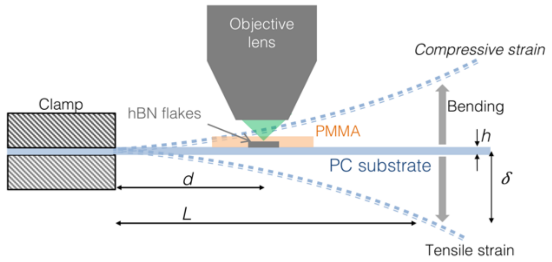

Supplementary Figure 12: **Schematic of the strain experiment setup:** The PC substrate is clamped tightly at one side and the other side is pushed downwards or pulled upwards to impress tensile or compressive strain, respectively. The amount of strain applied on the flakes can be calculated using Euler-Bernoulli beam equation, which depends on all the relevant quantities indicated in the figure.

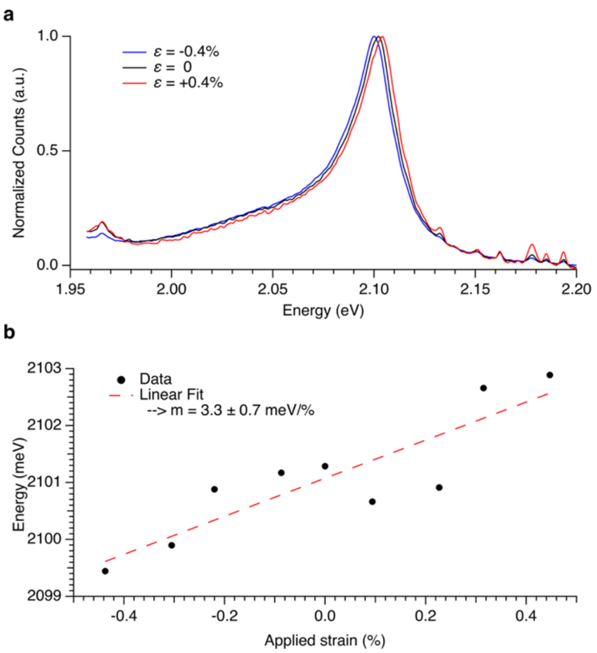

Supplementary Figure 13: **Spectral emission at different strain conditions:** ***a*** - Emission spectra of a single photon emitter for compressive (blue line), tensile (red line) and no strain (black line). ***b*** - Black dots show the central peak emission for different applied strain intensities. A linear fitting (dashed red line) yields a tunability of 3.3 meV/%

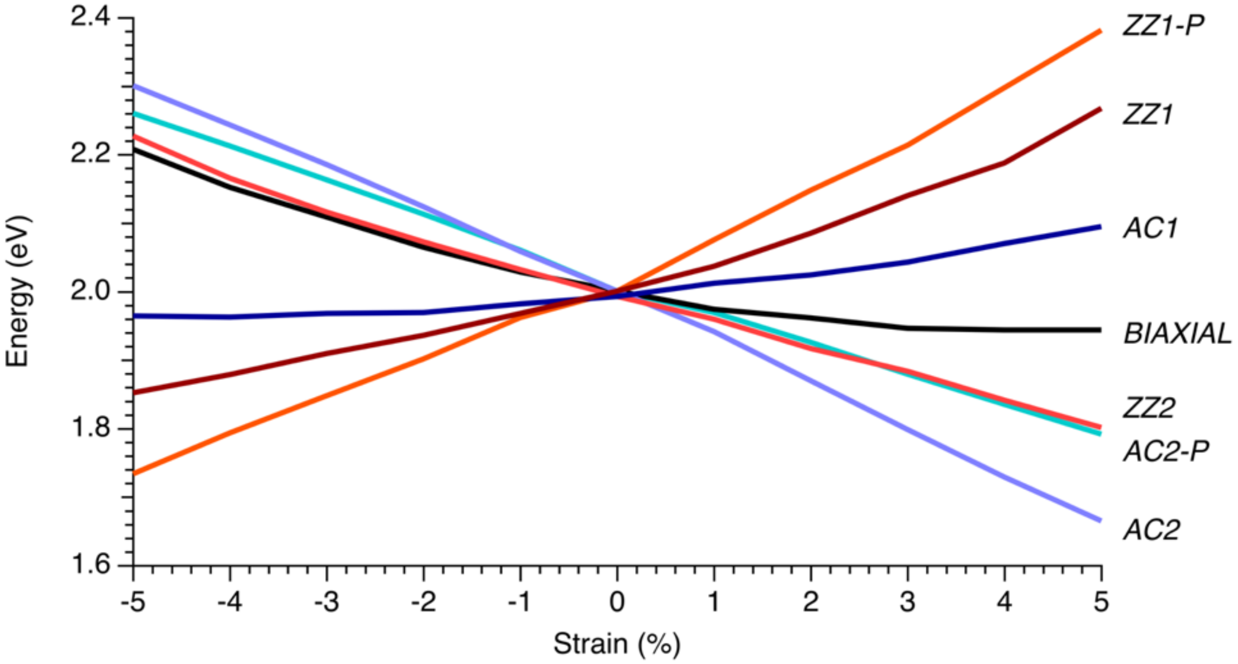

Supplementary Figure 14: **Simulated spectral emission as a function of applied strain:** The plot shows the simulated energy shift under strain applied along different directions. ZZ1, AC1, ZZ2, and AC2 mean uniaxial strain on each direction as labeled in inset of Fig.5. ZZ1-P and AC2-P show more realistic cases where the Poisson's ratio of the PC substrate is considered. In these cases, compression along AC2 (ZZ1) is induced when tensile strain is applied along ZZ1 (AC2). BIAXIAL is homogeneous biaxial strain along ZZ1 and AC2.
